# Supplementary material for: Asynchronous magnetic resonance elastography: Shear wave speed reconstruction using noise correlation of incoherent waves
Source: Magn Reson Med. 2022 Oct 27;89(3):990–1001. doi: 10.1002/mrm.29502 (PMC9792433; doi:10.1002/mrm.29502)
Supplement: Supplementary file 1 — DATA S1 MATLAB code used to generate the numerical simulations of Figure 1 [file MRM-89-990-s002.zip › k-Wave/helpfiles/k-wave_numerical_analysis.html]

Numerical Analysis (k-Wave)


# Numerical Analysis

- Example: Controlling The Absorbing Boundary Layer
- Example: Source Smoothing
- Example: Filtering A Delta Function Input Signal
- Example: Modelling Power Law Absorption
- Example: Modelling Nonlinear Wave Propagation
- Example: Optimising k-Wave Performance
